# Community-based hepatitis B screening: what works?

**DOI:** 10.1007/s12072-014-9562-4

**Published:** 2014-08-01

**Authors:** Monica C. Robotin, Jacob George

**Affiliations:** 1 Cancer Council NSW and University of Sydney, Sydney, NSW Australia; 2 Storr Liver Unit, Westmead Millennium Institute and Westmead Hospital, University of Sydney, Westmead, Sydney, NSW Australia

**Keywords:** Chronic hepatitis B, Hepatocellular cancer, Cancer screening and prevention, Community-based screening

## Abstract

**Introduction:**

Chronic hepatitis B (CHB) affects over 350 million people worldwide and can lead to life-threatening complications, including liver failure and hepatocellular cancer (HCC). Modern antiviral therapies could stem the rising tide of hepatitis B-related HCC, provided that individuals and populations at risk can be reliably identified through hepatitis B screening and appropriately linked to care. Opportunistic disease screening cannot deliver population-level outcomes, given the large number of undiagnosed people, but they may be achievable through well-organized and targeted community-based screening interventions.

**Material and methods:**

This review summarizes the experience with community-based CHB screening programs published in the English-language literature over the last 30 years.

**Results:**

They include experiences from Taiwan, the USA, The Netherlands, New Zealand, and Australia. Despite great variability in program setting and design, successful programs shared common features, including effective community engagement incorporating the target population’s cultural values and the ability to provide low-cost or free access to care, including antiviral treatment.

**Conclusion:**

While many questions still remain about the best funding mechanisms to ensure program sustainability and what the most effective strategies are to ensure program reach, linkage to care, and access to treatment, the evidence suggests scope for cautious optimism. A number of successful, large-scale initiatives in the USA, Asia–Pacific, and Europe demonstrated the feasibility of community-based interventions in effectively screening large numbers of people with CHB. By providing an effective mechanism for community outreach, scaling up these interventions could deliver population-level outcomes in liver cancer prevention relevant for many countries with a large burden of disease.

## Background

Chronic infection with hepatitis B virus represents a global public health challenge, given that approximately 350 million people are infected worldwide [1]. Approximately 95 % of infected adults and older children can successfully clear the infection and become immune, but 90 % of infected neonates and 25–50 % of children infected in infancy become chronically infected [2]. Chronic hepatitis B (CHB) can remain asymptomatic for decades, but can lead to cirrhosis or hepatitis B-related liver cancer (hepatocellular cancer, or HCC) in approximately 25 % of cases, explaining the 800,000 deaths/year attributable to the infection and its complications [3, 4]. The Global Burden of Disease study estimated that, of the 8.0 million lives lost to cancer in 2010, HCC was second only to lung cancer in terms of cancer deaths; half of these cases were hepatitis B related [4].

Over 80 % of liver cancers occur in East Asia and Sub-Saharan Africa [5]; with increasing international migration, increasingly they are also HCC disease determinants in North America, Western Europe, and Australia, particularly among immigrant populations [6–9]. US Vietnamese males are 11 times more likely to develop HCC than non-Hispanic Whites [10], and Australian males born in Vietnam are 13 times more likely to develop HCC than other Australians [8].

Currently available antiviral therapies have the potential to change the natural history of CHB, [11–14] given that screening and treating high-risk populations appear cost effective in studies from the USA [15], Canada [16], Australia [17], and The Netherlands [18]. This is predicated upon people being aware of their status and willing and able to access regular monitoring and treatment [19], not readily provided through opportunistic CHB screening. Current estimates suggest that two-thirds of Americans [19] and 40 % of Australians living with CHB [20] are unaware they are infected; in the European Union this figure may be as high as 90 % [21], with people undiagnosed (many of them migrants and underserved populations) destined to replicate the natural history of the disease [22].

Community-based screening could provide CHB screening in populations where limited English proficiency, lower socioeconomic and educational levels, lack of health insurance, and disease stigma preclude their ability to effectively navigate the health care system [23], with health care provider- and health system-related barriers posing additional challenges [24]. Hepatitis B vaccination is the mainstay of modern hepatitis B prevention. The implementation of universal vaccination has led to dramatic reductions in the overall hepatitis B disease burden, and as of July 2011, 179 countries reported inclusion of the hepatitis B vaccine in their national immunization schedules (up from 31 countries in 1992) [3]. However, vaccination is of no benefit to those already infected, who need to access medical care to mitigate disease outcomes [19]. Disease screening offers people already infected a gateway into care, which needs to remain open until the pool of existing infections is exhausted. While the approach to screening may vary, identifying those infected remains a priority in all countries which have sizable at-risk populations.

This systematic review examines the evidence around community-based hepatitis B screening, seeking to better understand the common factors of success and challenges.

## Methods

We used Rein’s definition of community-based hepatitis B screening programs, as those that “systematically offer HBsAg testing to all members of a population group based on country of birth or participation in high-risk behaviour.” This definition excludes “screening conducted by state and local public health departments, including screening performed by refugee health programs” [25].

Whitehead views community-based interventions (CBIs) as alternatives to “top-down” interventions designed to improve the health and/or socioeconomic status of the world’s poor [26]. Based upon who initiates, drives, and carries out the intervention, he proposes seven types of community-based interventions, ranging from completely self-sufficient programs, driven and funded exclusively by the community (type 1) to those planned and implemented as equitable partnerships by the community in collaboration with an external change agent (type 7). The continuum includes interventions involving the recipient community to different degrees, from merely program recipients to active partners in program implementation, with the “ideal” CBI being a true partnership between technical experts and the communities they serve. The former contributes conceptual strength, comprehensive design, and rigorous implementation, while community endorsement and support increase the likelihood of program incorporation into its sociocultural context, strengthening sustainability and diffusion [26].

We graded the effectiveness of community engagement as “high” or “low” according to the programs’ self-reported capacity to establish meaningful community partnerships.

Programs were also categorized using the four hepatitis B screening models described by Rein et al. [27] as:

1. Community clinic model (CCM), with screening integrated into routine primary care services; the screening decision is informed by risk factor review, with doctors providing counseling and testing referrals.
2. Community outreach model (COM), which involves screening in community settings (i.e., health fairs and community centers), with testing provided by phlebotomists and with volunteers providing logistical support at screening events.
3. Partnership and contract model (PCM), in which screening is contracted to general health screening companies (such as wellness campaigns targeting Asian employees).
4. Outreach and partnership model (OPM), which combines elements of COM and PCM; screening takes place in COM-type settings, with planning activities coordinated by a community organization with direct links to the target community.

**Fig. 1:**
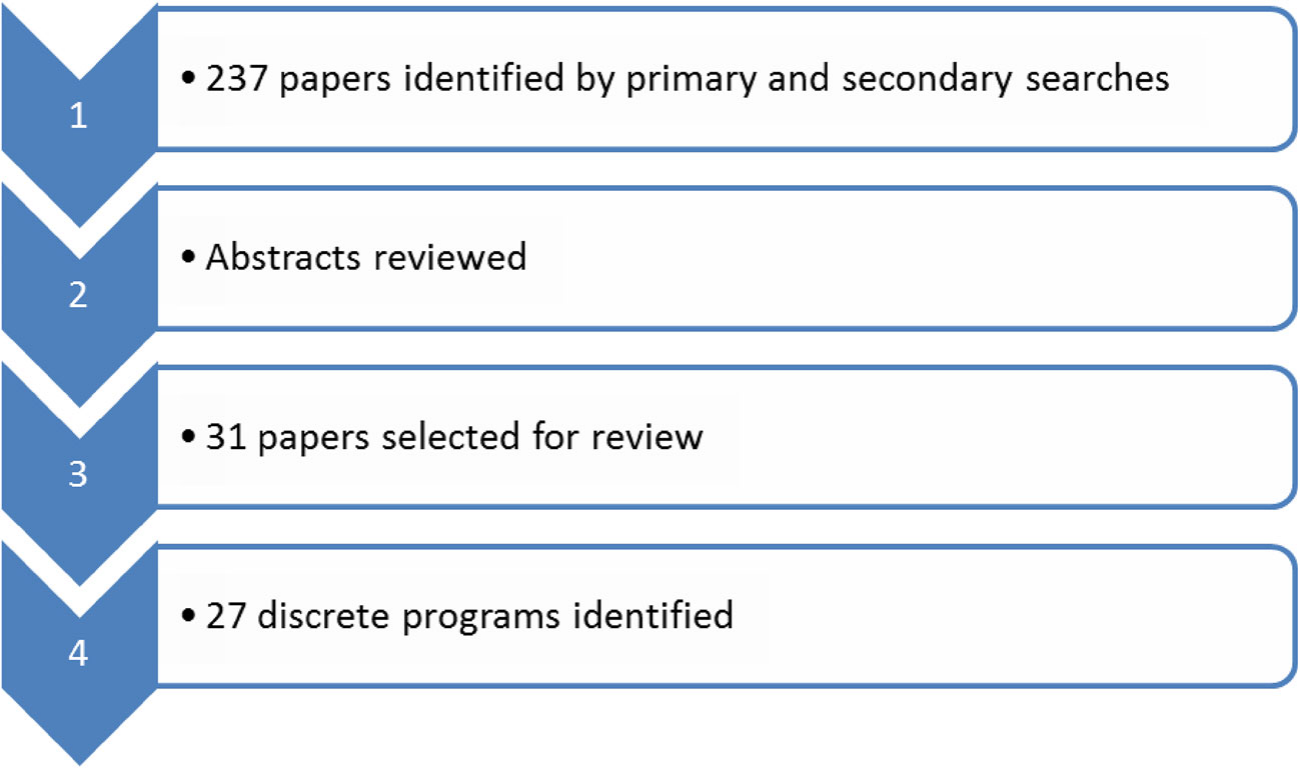
Diagrammatic representation of the search strategy and its outcomes

We identified publications about community screening programs by searching PubMed and EMBASE for articles published in the English language from 1984 through January 2014, using the terms “hepatitis B testing,” “hepatitis B screening,” combined with “community programs,” “migrant screening,” “CHB screening,” “high risk,” “population,” and “population-based screening.” Articles were entered into an Endnote (version X4, Thomson Reuters) database and identified abstracts reviewed. Full articles were retrieved if deemed relevant, with the list augmented with manual searches of reference lists. Where more than one publication described the same program, the paper providing the greatest level of detail was used as a key reference, with additional data from other publications included (and referenced) if they contributed salient information (i.e., updates on program outcomes). The overall search strategy is outlined in Fig. 1. Programs not providing details about how screening was conducted were excluded.

We extracted and tabulated the following information for each program:

1. Screening model employed and extent of community engagement
2. Program’s target population
3. Program partners
4. Study type
5. Program components and services provided
6. Program outcomes
7. Program costs

## Results

The search identified 237 papers; based upon the information provided in the abstracts, 206 papers were excluded, leaving 31 papers for review. As four of these reports described the same interventions (either different aspects or at different points in time), the final number of discrete programs was 27. Of these, 19 interventions were based in the USA, 4 in the Netherlands, 2 in New Zealand, and 1 each in Taiwan and Australia (Table 1).

**Table 1:** Hepatitis B screening programs identified by the literature search and their key features, components, and outcomes

Author	Project name/target population/aim/duration	Agent delivering intervention/mode of service delivery	Program components and services provided	Outcomes and recommendations	Community engagement extent (L/?/H) and model used
Milne et al. [28]	Population of Kawerau, North Island, New Zealand	Hepatitis Foundation (NGO) in Bay of Plenty, North Island, NZ	Hepatitis B screening Vaccination for those susceptible	7,901 people screened (93 % of the population of Kawerau)	? OPM
Hsu et al. [42]	Hepatitis B initiative: targeting AAPI in Boston Goals: educate, empower, and eradicate HBV in affected communities Reported activity from 1997 to 2002	Student volunteers from Harvard University’s Public Health and Medical Schools (and other local universities)	Awareness campaign using posters, info kits for local media and schools, talks, health fairs, radio, and “guerrilla media” events Free testing at community health center Free vaccination	997 free screenings; 39 % of susceptible offered free vaccination; 59 % completed 3 shots Strong and committed student leadership, annual recruitment and training of student volunteers Now also targeting African Americans	H COM
Lee et al. [30]	9Health fair Collected data on HBV status of AAPI migrants in Colorado in 2002 Aim: address high HCC rates in Korean and Vietnamese communities	Community partnerships with Korean and Vietnamese communities, the Asian Pacific Development Center, and Colorado Dept. of Health Hepatitis B testing at community health fair (9Health Fair)	Educational brochures distributed in churches, temples, and Korean stores Advertising: local media, posters Convenient testing sites and bilingual volunteers used Results mailed	Of 1,117 AAPI fair participants, 161 were screened; 7 (4.3 %) HBsAg +ve Identified a need for effective HBV prevention programs to reduce HCC incidence and health disparities	? PM
Herman [46] Robinson et al. [47]	HepBFree: NZ Hep B screening and follow-up program, ongoing Targeted 15–40-year-old Maori, Asians, and Pacific Islanders in Auckland and Northland regions of New Zealand	Hepatitis Foundation (community screening) and Northern Region Hepatitis Consortium (opportunistic GP screening) Screening in local facilities (*marae*), mobile caravans, and local GP offices	Ethnic specific outreach in community settings Hep B screening Follow-up and care Free vaccination Contact tracing	177,000 tested, 5.7 % HBsAg +ve; highest prevalence (13 %) in Tongans, 6.2 % in Asians, 5.6 % in Maori Low uptake (10 %) for GP model invitation letters Multiagency collaborations and culturally appropriate services needed to establish community trust	?H Multimodel
Chen et al. [29]	Measured HBV and HCV seroprevalence in Taiwanese aged ≥18 years Screening results 1996–2005	Liver Disease Prevention and Treatment Research Foundation, Taiwan Screening at “screening stations”	Limited details re community engagement: invites to attend screening stations by mail and via local media Tested liver function, anti-HCV, α-fetoprotein	164,302 screenings, 17.3 % HBsAg +ve; 4.4 % anti-HCV +ve Intercounty differences in prevalence rates observed	? OPM
Hsu et al. [40]	Aim: educate, test, and vaccinate local Asian population in Montgomery County, Maryland Oct 2005–July 2006	Partnership of 9 faith/community organizations, AAPI community, care providers, academic institutions, and local Dept. of Health and Human Services	Educational activities for care providers and local community reached via language schools, community centers, and health fairs Pre/posttest survey Free community screening Free vaccination	807 subjects from eight AAPI groups tested Highest infection rates in Cambodian (7 %) and Thai (7 %) % susceptibles highest in Asian Indians (70 %) and Thai (56 %) Infection rates and knowledge scores negatively correlated; targeted HBV education needed	? OPM
Marineau et al. [45]	Filipino community, Hawaii 2005–2006 One-off health fair	Key stakeholders from Filipino health care and church communities	Outreach via community media, churches, and grassroots effort Free blood tests for hepatitis B and C Abnormal results sent to individual’s health care provider	500 attended, 167 tested, 5 HBsAg +ve Knowledge gap re HBV transmission, risk factors, immunization Culturally sensitive interventions need to factor in language, cultural, and economic barriers to care	? COM
Juon [36]	Hepatitis B initiative-DC Targeting Asian American adults in Baltimore–Washington DC to prevent HCC 2003–2006	Piloted a faith-based HBV program with Korean church	Culturally and linguistically appropriate outreach materials Developed social support networks Provided HBV education Screening and vaccination events Offered technical assistance for other campaigns	1,775 people tested, 61 % susceptible (79 % completed 3-shot vaccine series), 2 % HBsAg +ve Culturally tailored booklets on HBV Integrating traditional beliefs in educational programs key factor for success Program extended to nine Korean and Chinese churches and via pastors’ conference	H OPM
Tipper and Penman [54] Robotin et al. [53]	B Positive Targeting Chinese- and Vietnamese-born Australians in SW Sydney 2007	Cancer Council NSW Partnership with local Division of General Practice, specialists, RACGP, community leaders, and associations CHB screening and F/U at GP surgeries	GP education CHB screening and F/U protocol Community awareness and education via ethnic media and events Economic modeling Disease registry	CHB screening and treatment found to be cost effective Poor initial results prompted extensive community and provider consultation 1,200 people enrolled in registry; community engagement key factor	L initially CCM
Chang et al. [38]	Three for Life Targeted foreign-born Chinese Americans in the Richmond District of San Francisco 2004–2005	Asian Liver Center and SF Department of Public Health Testing and vaccination at SF Richmond District YMCA	Free HBV testing Screening and subsidized vaccination Education using bilingual brochures	1,106 people tested; 9 % were HBsAg +ve, 53 % susceptible (85 % completed vaccination) Program replicated in LA, San Diego, Arizona, Hawaii	? OPM
Rein et al. [25]	Audit of US community-based programs offering systematic CHB screening based upon COB or high-risk behavior	Collected information on service delivery of CHB community screening	Collected information on location, services provided, groups targeted/HBsAg prevalence among those screened	55 possible programs identified, 31 reached; 21,817 screened in 1 year, 8.1 % HBsAg +ve Seroprevalence highest in Vietnamese (9.7 %), Chinese (8.0 %) 90 % of programs offered HBV screening and vaccination, 74 % HBV education, 71 % referrals, 29 % treatment	? Multimodel
Bailey et al. [31] Overall strategy and evaluation by Gish and Cooper [55]	San Francisco Hep B Free (SFHBF) Targeting API community in SF Aim: to make SF the first hepatitis B-free city in the USA Results detailed for 2007–2009	Grassroots, community-based health initiative Key players: Asian Liver Centre, SF Dept. of Public Health, API community, ethnic media, California Pacific Medical Center, and Sutter Pacific Medical Foundation	Culturally targeted awareness-raising promoting testing and vaccination Used ethnic media, brochures, Internet resources Offered free testing and low-cost vaccination Used bilingual hospital/clinic staff and volunteers	>400 community partners Engaged >150 organizations; reached 1,100 care providers and >200,000 people Providing care for uninsured challenging Comprehensive program evaluation included community impact	H OPM
Hwang et al. [43]	Aim: identify HBV and HCV prevalence among AAPIs and facilitate specialist referral rates in Houston, TX	One-off testing at community health fair Coalition of community and academic organizations	Testing advertised via newspapers, TV, community networks Hep B +ve people phoned and sent customized in-language letters and provided referrals	202 people screened, 118 AAPIs; 13.6 % had CHB; 92 % unaware of infection Successful referrals: 83 % for CHB, 100 % for HCV Recommended a population-based viral hepatitis registry	? COM
Lee et al. [41]	Part of Healthy Asian Americans Projects Aim: study HBV prevalence as baseline to devise education and interventions for AA in Michigan Duration: 2006–2008	Screened Chinese, Korean, Vietnamese AAPI at community fairs Program delivered by University of Michigan in collaboration with local community and health service organizations	Advertised via flyers, health fairs, community media Free HBV screening for HBV surface Ag and Ab Provided community education through 30 articles in ethnic media and brochures translated into six languages	567 participants tested at 8 health fairs; screening rates 36–94 % ~6 % had CHB, 40 % susceptible >95 % migrants, 45 % without health insurance Recommended language-specific, culturally sensitive educational interventions	? OPM
Sheu et al. [56]	San Francisco Hepatitis B Collaborative (SFHBC) Targeting APIs in San Francisco 2004–2009	Focused health disparity curriculum developed by students at UCSF and aligned with SFHBF and Department of Public Health efforts	Recruitment via language-concordant media, email, provider referrals, community presentations Student clinics offered free screening and low-cost vaccinations/referrals	477 students educated and screened; 804 participants from 14 countries 63 % participants had limited English proficiency, 55 % had annual household income <25,000 USD; 46 % were uninsured 10 % HBsAg +ve, 44 % susceptible	? CCM
Chao and So [32] Early results described by Lin [57]	Jade Ribbon Campaign (JRC) Targeting AAPI in San Francisco 2001–2004 Aim: raise disease awareness and promote screening	Asian Liver Center working with >400 community partners Together with SF Department of Public Health and Chinese media, formed the basis for the SFHBF campaign	Raised awareness among AAPI and health professionals Provided access to vaccination and incorporated API values in program Outreach: ethnic media, educational brochures, and web-based resources Disease advocacy	Screened 12,308 people; 85 % vaccine completion rate Recommended screening second-generation AAPIs Program: national hepatitis B model, precursor of San Francisco Hep B Free campaign	H OPM
Kallman et al. [48]	Aim: HBV, HCV prevalence in a Vietnamese community in Virginia	Testing at a local doctor’s office and annual Vietnamese health fair	Demographic and clinical data collected No educational component described	322 Vietnamese tested: 2.2 % anti-HCV +ve, 9.3 % HBsAg +ve Overall low HBV vaccination rates Suggested HBV testing by risk factor profile, not abnormal LFTs	? Multimodel
Pollack [34] Trinh-Shevrin [35]	Aim: to promote screening and access to Rx in Chinese and Korean Americans; BfreeNYK targeted also other nationalities at higher risk The New York City pilot program: 2004–2008	Coalition-driven initiative (five key partners) driving comprehensive effort to decrease HBV disparities in Asian American (AA) community Engaged health provider organizations, Department of Health, NY University	Community outreach and education Multimedia campaign in ethnic media Educational website Free screening and vaccination Screening and F/U using standardized protocols Advocacy work	Screened 9,000 people; 18 % tested +ve, 57 % linked to care Findings informed CDC HBV screening guidelines Costs per participant: screen and vaccinate, 273 USD; education outreach, 139 USD; 1,344 USD/year/infected case Now funded as a National Center of Excellence	H OPM
Rein et al. [27]	Describes outcomes of a specific pilot program funding community-based hepatitis B screening programs July 2008–Jan 2009	Screening and program data collected from five funded programs to identify different models of service delivery, demographic data on those screened, and cost/screen	Screening models: community clinic (CCM): community outreach (COM) partnership and contract (PCM) outreach and partnership (OPM); community screening supported by community organization	Programs screened 1,623 participants; 54.2 % without insurance/regular Dr CCM program screened fewest participants with cost/screen 40 USD; PCM screened most with cost 280 USD Best to identify populations amenable to clinical versus community outreach	N/A All models
Richter et al. [50]	Testing of Turkish residents of Arnhem, The Netherlands for hepatitis B and C 2008 onwards?	Local hospital’s infectious disease unit, migrant resource center, Turkish GPs, and Municipal Public Health Service	Customized resources: poster, brochure, video, website, and hotline Advertising via ethnic media, mosques, Turkish businesses F/U: own GP and hospital clinics Counseling and contact tracing	15 educational meetings, 450 participants 709 people screened, 18 with CHB, 2 with active HCV infection Screening process cumbersome; suggested integrating screening into routine clinical care	? Multimodel
Ma et al. [39]	Church-based HBV screening and vaccination program for Korean communities in Philadelphia and New Jersey	Center for Asian Health (CAH) at Temple University and the Asian Community Health Coalition (ACHC): academic–community partnerships Goals: increase HBV knowledge and awareness, screening and vaccination, and health care utilization in CHB	Community-based participatory research, and delayed HBV intervention in controls Pilot: 2 churches in intervention, 2 as controls Low-cost HBV test, vaccination, and consultation Health care providers offered patient navigation	330 participants; flexible clinic hours Significant increase in HBV screening in intervention group Challenges: financial constraints, access for under/uninsured, limited English proficiency Subsequently awarded 5-year grant to implement a full-scale program in 30 Korean churches in PA and NJ	H OPM
Veldhuijzen et al. [49]	Campaign targeting Chinese community in Rotterdam 2009	Rotterdam Municipal Public Health Service, Erasmus Medical Center, and National Hepatitis Center	Disease awareness activities through outreach Knowledge testing Free HBV testing at outreach locations Guideline-based specialist referral	1,090 Chinese migrants tested; 8.5 % (92) HBsAg +ve, 38 % referred to specialists; 15 started antivirals A convenience sample answered before–after knowledge questions; found improved knowledge score postintervention	? Multimodel
Perumalswami et al. [37]	Hepatitis Outreach Network (HONE) Targeting foreign-born individuals at risk of hepatitis B or C in NYC 2009–2011	Collaboration between Mt. Sinai Med School, NYC DoH, and CBOs	Publicity (radio, TV, PSA, papers) Community education Screening at community events Free vaccination Linkage to care using patient navigators	1,603 people educated and screened at 25 events involving participants born in 68 countries 76 diagnosed with CHB, 75 with HCV Success factors: engaging CBOs, publicize events, relevant languages, and patient navigators	H OPM
Van der Veen et al. [51]	RCT in Turkish migrants aged 16–65 in The Netherlands	Rotterdam Municipal Public Health Service, Erasmus Medical Center, University Medical Center	Culturally tailored intervention via the Internet Participants assigned to: BCT (behaviorally and culturally tailored)/BT (behaviorally targeted) or GI (generic info) arms Free HBV screening offered in each arm	10,069 persons invited, 1,512 (15 %) logged onto the website, 623 tested Screening uptake was 44, 46, and 44 % per arm BCT had favorable intervention effects, but no added value on screening uptake compared with BT	? CCM
Woo et al. [44]	Testing for hepatitis B (year 1) and B and C (year 2) by a single center at a community fair over 2 years	Schiff Center for Liver Diseases, Miami University, FL Free screening offered to all Asian Culture Festival participants aged 18–65	Free screening for hep B and C provided by multilingual Schiff Center staff ? information/education +ve tests mailed results and F/U phone calls made	Year 1: 1.6 % (173) attendees tested (31 % Asian descent), 1 HBsAg +ve; year 2: 2.6 % (231) tested (22 % of Asian descent); 3 HBsAg +ve 50 % HBsAg +ve contactable for F/U Screening incentives ineffective	? COM
Xu et al. [33]	Targeting Korean and Chinese American communities in LA County 2007–2010	Asian Pacific Liver Center (APLC) in LA: not-for-profit organization providing community outreach	Free screening events advertised in ethnic media and places of worship Lectures on CHB; test results mailed HBsAg +ve were encouraged to get medical F/U Comprehensive work-up if seeing specialists	7,387 people screened (93 % Korean/Chinese) at 63 events CHB prevalence 5.2 %; 99 % of 387 +ve born overseas, 22 % spoke no English; 26 % were insured Most F/U if insured (57 %) and having active disease	? OPM
Zuure et al. [52]	Aim: to investigate prevalence and determinants of HCV and HBV infection in Egyptian FGM in Amsterdam 2009–2010	All Egyptian organizations in the Amsterdam area contacted and KOL enlisted Public Health Service of Amsterdam (PHSA)	Viral hepatitis educational sessions delivered by Arabic educators Free screening sessions at Egyptian meeting places and PHSA Infected participants referred for F/U	11 educational and screening sessions; 465 people tested HBsAg +ve 1.1 %, all genotype D 2.4 % HCV Ab +ve Risk factors: older age + parenteral antischistosomal therapy	? Multimodel

*AAPI* Asian Americans and Pacific Islanders, *F/U* follow-up, *FGM* first-generation migrants, *HBV* hepatitis B virus, *HCV* hepatitis C virus, *HBsAg* hepatitis B antigen, *CBOs* community-based organizations, *CCM* community clinic model, *COM* community outreach model, *PCM* partnership and contract model, *OPM* outreach and partnership model

Two US papers reported aggregate results of US-based community screening programs: one reported outcomes of a nationwide audit of community-based hepatitis B screening programs [25]; the other described four models of community-based screening [27], which we also used for consistency.

### Screening model employed and estimated degree of community engagement

An OPM was employed by 13 programs. Some were large one-off initiatives (e.g., screening the entire population of Kawerau, New Zealand [28], the adult population of Taiwan [29], the Asian American and Pacific Islander migrants in Colorado, USA [30]), while others operated for a longer duration, such as programs in California (Hep B Free [31] and the Jade Ribbon Campaign in San Francisco [32] and a program run by the Asian Liver Center in Los Angeles [33]) and the BFreeNYC program in New York [34, 35]. Medium-sized OPM programs screened 1,000–2,000 participants: the Hepatitis B Initiative in Washington, DC [36], the Hepatitis Outreach Network (HONE) program in New York [37], and the Three for Life initiative in San Francisco [38]. Smaller OPM programs (screening <1,000 people) were run in conjunction with faith-based community organizations (i.e., Korean churches in New Jersey [39] and Montgomery County in Maryland [40]) and through health fairs in Michigan [41]. In addition to hepatitis B screening, OPM programs included specific outreach and educational activities, including hepatitis talks, distribution of printed materials, and web-based resources and effectively used ethnic media for publicity.

COMs provided screening through one-off events at community health fairs and/or community centers. All were US based and targeted Asian Americans and Pacific Islanders in Boston [42], Houston [43], Miami [44], and Hawaii [45]. No ongoing community engagement was documented, and they reached between 100 [45] and 1,000 people [42].

The HepBFree program in New Zealand used community screening with outreach in rural areas and screening in general practices (GPs) in Auckland [46, 47]; the latter was also employed by a program in Virginia, which combined testing at a local doctor’s surgery with testing at an annual fair [48].

Multiple methods were employed by the Dutch initiatives: testing was offered in community centers, schools, churches, and the Municipal Public Health Service in Rotterdam and Arnhem [49, 50]; an Internet intervention was trialled in Rotterdam [51], and screening at Egyptian meeting places and the Public Health Service was offered in Amsterdam [52].

In San Francisco, clinic-based screening was offered by the Three for Life program [38] and through clinics run by medical students. The Australian program offers primary care-based screening by GPs in Sydney [53, 54].

Sufficient information allowed us to ascertain a high degree of community involvement in eight programs; the Australian B Positive program commenced as a clinical intervention delivered by general practitioners and was repositioned as a community–agency collaboration to increase program visibility and participation rates [53].

### Program target population

The target populations ranged from country-wide hepatitis B and C screening in Taiwan [29] to city-wide screening in New York (BFreeNYC [34]) and San Francisco (Hep B Free) programs [31]. Screening targeted people of Asian and/or Pacific Islander heritage in Boston [42] and Maryland [39, 40] and the HONE program in New York [37]. The HepBFree program in New Zealand targeted the local Maori population, as well as Asian and Pacific Islander residents [46, 47]. Korean and Vietnamese Americans were the target population in Colorado [30], Korean and Chinese Americans in the Baltimore–Washington area, LA County, and San Francisco [31, 33], Chinese, Korean, and Vietnamese Americans in Michigan [41], the Filipino community in Hawaii [45], and Chinese–Korean communities in Philadelphia and New Jersey [39]. In Australia, the B Positive program targets Chinese and Vietnamese residents in Sydney [51], while Dutch programs targeted Chinese and Turkish migrant communities of Rotterdam and Arnhem [49–51], and Egyptian migrants in Amsterdam [52].

Some US-based programs were promoted and supported by faith-based organizations [36, 39, 40], and some were offered by clinical groups offering education and testing at community events [30, 43, 45]; while some screened all participants (in Miami, FL and Houston, TX) [43, 44], others based testing decision on risk factors (Colorado) [30].

In New Zealand, testing was offered at Maori meeting places (*marae*), mobile caravans, and through GP offices [46]. In Australia, it is offered through GP offices [54], and in The Netherlands at community sites and Municipal Public Health Services [49–52]. The Taiwanese program invited participants to attend clinics at designated screening stations [29].

### Program partners

Most programs were the result of collaborations between academic institutions or clinics and community-based organizations; some also had support from local public health units. The number of community partners ranged from >400 in the case of San Francisco Hep B Free [32] to just the agency delivering the intervention [41, 44].

### Study type

Two reports described controlled intervention studies: one was a church-based HBV screening and vaccination pilot program in Philadelphia [39], the other a randomized controlled trial (RCT) conducted in The Netherlands [51].

The US pilot study recruited 330 Korean Americans through churches in the intervention area, and randomized them to either HBV education and HBV testing at enrollment (the intervention group), or to a delayed intervention, where these services could be accessed at a later stage (the control group). A statistically significant increase in HBV screening was observed in the early intervention group compared with controls [39].

The Dutch study recruited first-generation Turkish residents of Rotterdam to a culturally tailored Internet-based intervention aiming to promote HBV screening [51]. Through a clustered randomized design, participants were computer-randomized to receive either a behavioral tailoring intervention (BT), one combining behavioral and cultural tailoring, or just generic online information. An invitation letter explained the intervention and directed recipients to the project’s website, which “streamed” participants into one of the three intervention groups. Approximately 15 % of those sent letters logged onto the website, and overall screening uptake was similar (~45 %) across all three intervention groups [51]. This was the first documented intervention using the Internet to increase hepatitis B testing rates in a migrant community; given the low participation rate, these findings need further validation [51].

The remaining 25 papers describe nonrandomized screening interventions which incorporated some form of community outreach and education in addition to screening.

### Program components and services provided

Programs publicized hepatitis B screening using ethnic media and flyers/posters; all but 3 (88 %) offered community education using lectures and workshops, educational brochures, articles published in ethnic newspapers, and web-based resources. City-wide programs in San Francisco and New York had sophisticated multimedia campaigns and marketing strategies and developed program-specific websites with tailored educational information.

Vaccination (either free of charge or subsidized) was offered by 12 programs (48 %); most US programs and the New Zealand programs offered it. Vaccination was not included in the Dutch, Taiwanese, and Australian programs, which may be due to the ability to access vaccination through other means.

One-year follow-up was provided by the two controlled intervention studies, with the San Francisco Hep B Free [55] and the BFreeNYC [34] programs also providing follow-up, constrained by limited resources. Long-term follow-up is offered by the New Zealand [47] and Australian programs [54].

The Dutch [49–52], Australian [54], and New Zealand programs [46] as well as some US programs offered linkage to care [35, 43, 56] or employed a patient navigator to negotiate the medical system on the patients’ behalf [41, 57]. Programs in Michigan [41], Texas [43], Virginia [48], Florida [44], and Southern California offered referrals to insured participants [33]; 71 % of the US programs identified by Rein et al. [25] provided treatment referrals, with 29 % providing antiviral treatment.

A complete CHB care package encompassing hepatitis B screening, HCC surveillance, ongoing disease monitoring, and treatment was offered by BFreeNYC [34] and San Francisco Hepatitis B Free [55] and programs in New Zealand [46, 47], Australia [54], and The Netherlands [49].

Some programs provided hepatitis C testing [29, 37, 43, 45, 50, 52], contact tracing (the New Zealand program) [45] or physician education about HBV (some US and the Australian program) [32, 34, 54] or disease advocacy.

San Francisco seeks to become the first HBV-free city, with the Hep B Free Campaign offering screening, vaccination, and treatment to all Asian and Pacific Islander residents (representing 30 % of its population) [10]. To improve disease surveillance, the city established a population-based chronic hepatitis B registry, with enhanced disease surveillance ascertaining transmission patterns and participants’ ability to access hepatitis care [58]. The Australian program includes a CHB disease registry to optimize patient follow-up and collect population-level data on CHB disease characteristics [53, 54].

### Program outcomes

Most interventions reported results in terms of the number of people reached, number of screenings performed, and estimated HBsAg prevalence overall and by ethnic groups.

The most comprehensive outcome measures were documented by the BFreeNYC program, which also conducted a random survey of Asian Americans 2 years after the program ended [34]. They documented a 34 % increase in new CHB cases reported from areas with a high Asian population during its 4 years of activity, with 57 % of people with CHB remaining in care until the end of the program [34]. BFreeNYC reached over 1 million people, provided education for 11,000, screened approximately 9,000 people, and diagnosed and managed 6 cases of HCC and 22 of end-stage liver failure [34].

During its first 2 years, the San Francisco Hep B Free program reached over 200,000 people and tested 3,315 Asian–Pacific Islanders at standalone screening sites [31] and 12,000 people through the Jade Ribbon Campaign [32]; 6.5 % were chronically infected and referred for follow-up care [31]. The largest “yield” of screening occurred in higher education establishments with a large proportion of Asian students, Asian street festivals and fairs [55].

The HepBFree New Zealand program tested 177,000 people, 5.7 % being HBsAg-positive; significant regional and ethnic differences in HBsAg-positive rates were observed among Maori (5.6 %), Pacific islander (7.3 %), and Asian people (6.2 %) [47]. Successful outreach raised CHB community awareness and led to effective partnerships with local health care providers [47, 59].

With few exceptions, programs did not report the size of their target population, but the Kawerau study in New Zealand was able to test 93 % of the town population, finding HBsAg prevalence rates of 4.2 % among European residents and 18.2 % amongst the Maori population [28].

Rein et al. [25] reported results for five US screening programs screening over 1,600 participants over 7 months; 95 % of those screened were foreign-born, and most (56 %) did not have a regular medical practitioner or health insurance (54 %).

Screening uptake was highest for programs using an outreach and partnership model (OPM) [31, 33, 37, 59]; the COM at community fairs yielded fewer screenings [30, 34, 45]; screening offered by clinical experts had low uptake. The Healthy Asian American Projects initiative in Michigan targeted Chinese, Korean, and Vietnamese Asian Americans at eight health fairs over 2 years; despite wide advertising, education, and distribution of brochures in six languages, screening rates remained low, attributed to “resistance by Asian Americans to participate in clinical studies” [41]. Similar outcomes were documented by a program in Florida, where free access to specialists and a screening incentive led to 1.6 and 2.6 % of participants taking up screening in the first and second year, respectively [44].

Successful completion of hepatitis B vaccination was monitored by the Hepatitis B initiative in Boston (59 %) [40] and Washington (79 %) [36], as well as the Three for Life (85 %) [37] and Jade Ribbon campaigns in San Francisco [32].

Linkage to care (beyond vaccination) was offered by 11 programs, mostly in countries with socialized medicine: in Europe 2 (or 66 %) out of 3 (or 66 %) and in Australia–New Zealand 2 (or 66 %) out of 3 (or 66 %) programs offered linkage to care, compared with the USA, where 6 (30 %) out of 20 did so. In five US screening programs, 54 % of participants had no insurance cover and/or no regular health care provider [25]; in Michigan 45 % [41], in San Francisco 46 % [31], and in Los Angeles 74 % [33] of people accessing the programs were uninsured.

BFreeNYK was able to maintain 57 % of its 1,100 CHB patients in care until the end of the 4-year program [34], but high rates of loss to follow-up occurred in other programs: just 77 % of the 7,000 people screened by the Asian Pacific Liver Center in Los Angeles could be traced 6 months later [33].

## Program costs

Cost of care estimates were provided by the BFreeNYC program, with annual cost per infected patient estimated at 1,598 USD [34]. Rein et al. [27] compared the costs of four types of community screening in the USA and found that CCM was the least costly per screened participant, albeit screening fewer participants, while the partnership and contract model (PCM) screened most participants, at the highest cost per screening.

## Discussion

Over the last 30 years, many initiatives have sought to increase hepatitis B screening rates in high-risk communities, by targeting migrant populations in the USA, Australia, and The Netherlands, as well as indigent populations in New Zealand and Taiwan. A few programs successfully reached large numbers of people, but the majority screened modest numbers: the 31 programs active across the USA in 2008 screened a total of 21,817 people, or approximately 700 people per program. Even assuming seroprevalence rates of 10 % in the target populations, this translates into just 2,000 new CHB diagnoses. Given that the USA has approximately 2 million infected people [60], of whom 60 % (i.e., 1.2 million) are unaware of their infection [19], opportunistic screening cannot make a significant impact in populations with low access to medical care [19], making community-based screening a more attractive option. Successful programs achieved significant buy-in from target communities, delivering culturally appropriate educational initiatives and offering comprehensive care packages, as exemplified by the BFreeNYC [34], San Francisco Hep B Free [55], and the New Zealand [46, 47] and Australian programs [53, 54].

Large US programs grappled with the challenge of offering ongoing care to uninsured participants, as two-thirds of people not attending follow-up arrangements had no financial means or medical insurance [33]. The BFreeNYC program was the only US program able to provide free treatment over its 4-year existence [34]; the San Francisco programs faced great logistical challenges to provide access to care to uninsured [32]. Availability of free medical care did not ensure successful referral to care: one-third of patients eligible for treatment in a Dutch study did not see a specialist [61], and the uptake of the Sydney-based program was low initially, despite providing free screening and treatment [50].

Successful programs found innovative ways to leverage organizational and individual resources, including garnering political and practical support [34, 62]. To ensure program sustainability, costs and outcomes require close scrutiny; while CHB screening integrated with primary care services is less labor intensive and less costly, evidence from the USA [27] and New Zealand [47] suggests it delivers lower screening rates. Conversely, outreach models deliver greater community involvement, but at higher costs. The New York program suggested main-streaming these activities into primary care and educating primary care providers [34].

Key program challenges included the high cost of screening and limited ability to offer affordable long-term care, so new approaches and financing arrangements are critical to make access to care a reality for many. Most US programs relied upon volunteer support and commitment from communsity-based organizations, and reliance on their continued support may be unsustainable in the long run [34, 55]. Given that low community awareness, widespread misinformation, and persisting cultural stigma remain significant barriers, sustained community awareness-raising campaigns, complemented by culturally appropriate care delivery models, are acutely needed [24].

The noted “resistance by Asian Americans to participate in clinical studies” [41] prompted recommendations for educational interventions to be developed in native Asian languages, rather than using translated English resources [41]. Although previous research suggested that Asian Americans prefer to access health information from health care providers speaking their language [63], programs providing access to health specialists speaking Asian languages and offering screening incentives did not achieve a great deal of success [34].

The linkage to care and treatment is critical to ensure program buy-in and effectiveness, and this poses serious challenges in many countries with high CHB disease prevalence, but with costs of antiviral therapies likely to fall in the future, a community-based model of CHB diagnosis could still provide the impetus for offering a large-scale treatment program for a larger population.

Box 1 provides some summary points of critical success factors and program limitations and challenges.

**Box 1:** Factors ensuring effective program delivery

Community awareness and education
Using community networks and grassroots work to promote programs
Ethnic and language-specific program promotion
Maintaining an ongoing awareness campaign
Culturally and linguistically tailored outreach materials
Making effective use of ethnic media to publicize events and resources
Screening models incorporating community outreach
Bilingual or culturally aware staff delivering intervention
Offering flexible and varied screening options at suitable times and places
Developing and implementing standardized screening and follow-up procedures
Useful “add-ons”
CHB monitoring and treatment protocols integrated with medical records
Integrating CHB screening into routine care
Health provider education, training, and support
Access to patient navigators to provide linkages and patient assistance
Political endorsement and support
Advocacy at local and national level
On the “wish list”
Ability to provide affordable linkage to care, including ongoing disease monitoring and treatment
Large and renewable volunteer pool (or ideally funding for staff)
Disease register to facilitate follow-up and epidemiological data collection

## Conclusions

This review suggests that community-based hepatitis B screening is an active area of research and experimentation in countries with large migrant populations, such as the USA, The Netherlands, New Zealand, and Australia. Successful programs used a range of strategies to increase community awareness and knowledge and leveraged community partnerships to achieve significant community engagement and penetration. They combined HBV education, community empowerment, and collaborative partnerships, and they incorporated the target population’s values in program design and implementation. In addition to screening and vaccination, “ideal” programs must offer access to ongoing care and support, inclusive of antiviral therapy and HCC screening.

Many unanswered questions still remain regarding optimal funding mechanisms, program sustainability, the best way of ensuring linkage to care, and how to develop, select, and implement the most effective strategies of screening, disease surveillance, and community engagement and education.
